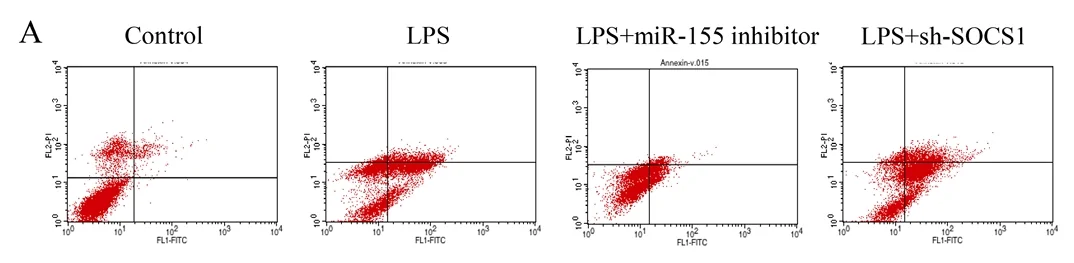# Corrigendum: Down-regulation of microRNA-155 suppressed *Candida albicans* induced acute lung injury by activating SOCS1 and inhibiting inflammation response

**DOI:** 10.71150/jm.2606100

**Published:** 2026-06-19

**Authors:** Xiaohua Li, Yuanzhong Gong, Xin Lin, Qiong Lin, Jianxiong Luo, Tianxing Yu, Junping Xu, Lifang Chen, Liyu Xu, Ying Hu

**Affiliations:** 1 Department of Pulmonary and Critical Care Medicine, Affiliated Fuzhou First Hospital of Fujian Medical University, Fuzhou, Fujian 350009, P. R. China; 2 Department of Pulmonary and Critical Care Medicine, 900 Hospital of the Joint Logistics Team, Fuzhou, Fujian 350001, P. R. China; 3 Department of Respiratory Medicine, Nanping First Hospital Affiliated to Fujian Medical University Nanping City, Fujian 353006, P. R. China

Corrigendum: Journal of Microbiology. 2022. 60: 402–410.

https://doi.org/10.1007/s12275-022-1663-5

­

The original online version of this article was revised:

This article contained errors in Figs. 2A and 5A due to incorrect image selection during fig. preparation. The hematoxylin and eosin (HE) staining image for the Control group in Fig. 2A and the flow cytometry image for the LPS+miR-155 inhibitor group in Fig. 5A were mistakenly selected from other groups within the same study project. These corrections do not affect the results, interpretation, or conclusions of the article. 

## Results

### Published Fig. 2A

The HE staining image for the Control group was incorrectly presented.

[Fig f1-jm-2606100]

### Corrected Fig. 2A

The correct HE staining image for the Control group is shown below.

[Fig f2-jm-2606100]

### Published Fig. 5A

The flow cytometry image for the LPS+miR-155 inhibitor group was incorrectly presented.

[Fig f3-jm-2606100]

### Corrected Fig. 5A

The correct flow cytometry image for the LPS+miR-155 inhibitor group is shown below.

[Fig f4-jm-2606100]

## Figures and Tables

**Figure f1-jm-2606100:**
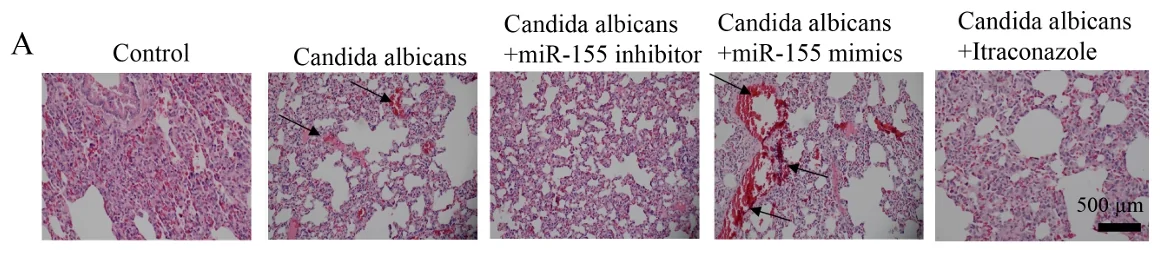


**Figure f2-jm-2606100:**
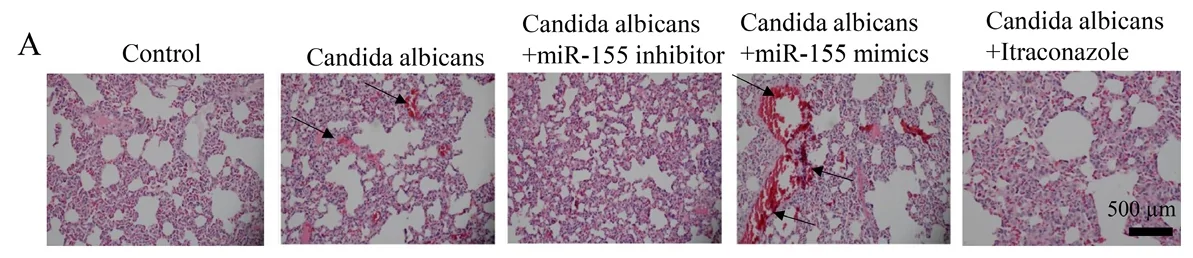


**Figure f3-jm-2606100:**
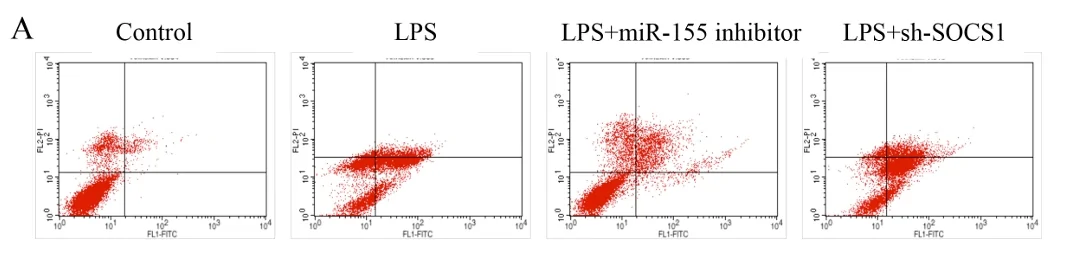


**Figure f4-jm-2606100:**